# Synergistic effect of bimetallic Pd–Pt nanocrystals for highly efficient methanol oxidation electrocatalysts†

**DOI:** 10.1039/d3ra04837c

**Published:** 2023-09-08

**Authors:** Respati K. Pramadewandaru, Young Wook Lee, Jong Wook Hong

**Affiliations:** a Department of Chemistry, University of Ulsan Ulsan 44776 Republic of Korea jwhong@ulsan.ac.kr; b Department of Education Chemistry and Research Institute of Natural Sciences, Gyeongsang National University Jinju 52828 Republic of Korea lyw2020@gnu.ac.kr

## Abstract

Metal nanocrystals (NCs) with controlled compositional and distributional structures have gained increasing attention due to their unique properties and broad applications, particularly in fuel cell systems. However, despite the significant importance of composition in metal NCs and their electrocatalytic behavior, comprehensive investigations into the relationship between atomic distribution and electrocatalytic activity remain scarce. In this study, we present the development of four types of nanocubes with similar sizes and controlled compositions (Pd–Pt alloy, Pd@Pt core–shell, Pd, and Pt) to investigate their influence on electrocatalytic performance for methanol oxidation reaction (MOR). The electrocatalytic activity and stability of these nanocubes exhibited variations based on their compositional structures, potentially affecting the interaction between the surface-active sites of the nanocrystals and reactive molecules. As a result, leveraging the synergistic effect of their alloy nanostructure, the Pd–Pt alloy nanocubes exhibited exceptional performance in MOR, surpassing the catalytic activity of other nanocubes, including Pd@Pt core–shell nanocubes, monometallic Pd and Pt nanocubes, as well as commercial Pd/C and Pt/C catalysts.

## Abstract 2

Nanocubes (Pd–Pt alloy, Pd@Pt core–shell, Pd, and Pt) were employed as electrocatalysts for methanol oxidation reaction to explore the correlation between atomic distribution within electrocatalysts and their catalytic activity.

## Graphical abstract

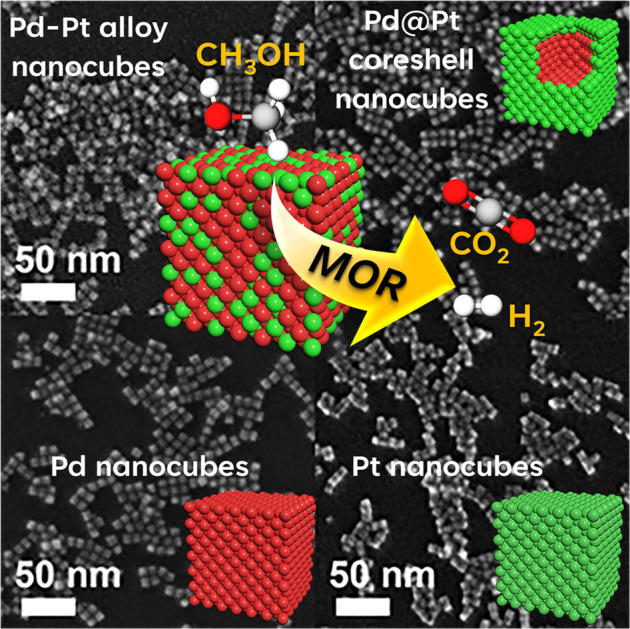

## 1 Introduction

Over the past few decades, there has been significant focus on high-performance fuel cells, which efficiently convert chemical energy into electrical energy while avoiding the emission of harmful gases.^1–6^ Among the various fuel cell types, direct methanol fuel cells (DMFCs) have garnered attention due to their exceptional energy density, high conversion efficiency, light-weight nature, and comparatively low operating temperature.^7–9^ Nevertheless, the slow kinetics of anodic oxidation and surface CO poisoning hinder the practical implementation of DMFCs.^10,11^ As a result, extensive research efforts have been initiated in this field.

Pt is widely recognized as one of the primary electrocatalysts utilized in various applications.^12–14^ To pave the way for the eventual large-scale commercialization of fuel cells, significant attention has been dedicated to advancing Pt-based materials, aiming to develop cost-effective, highly efficient, and long-lasting electrocatalysts.^15–17^ Currently, efforts are being directed towards addressing catalytic activity by means of controlling factors such as surface structure and composition.^18–27^ Among the various strategies, the control of compositional structure in Pt-based bimetallic nanocrystals (NCs) has emerged as a highly effective strategy for manipulating their electronic and geometric properties.^28–30^ This approach enables the optimization of the catalytic active centres, leading to the modification of adsorption/desorption properties of the intermediates involved in the methanol oxidation reaction (MOR).^31–33^ Among various metals for the formation of Pt-based bimetallic NCs, Pd is recognized for its capability to augment the MOR activity and durability of Pt-based catalysts.^34–37^ Given this background, Pd–Pt bimetallic NCs are widely regarded as highly promising candidates for efficient electrocatalysis in the MOR. However, the preparation of Pd–Pt bimetallic NCs with precisely controlled compositional structures and the in-depth investigation of their MOR properties have been rarely explored to date.

**Fig. 1:**
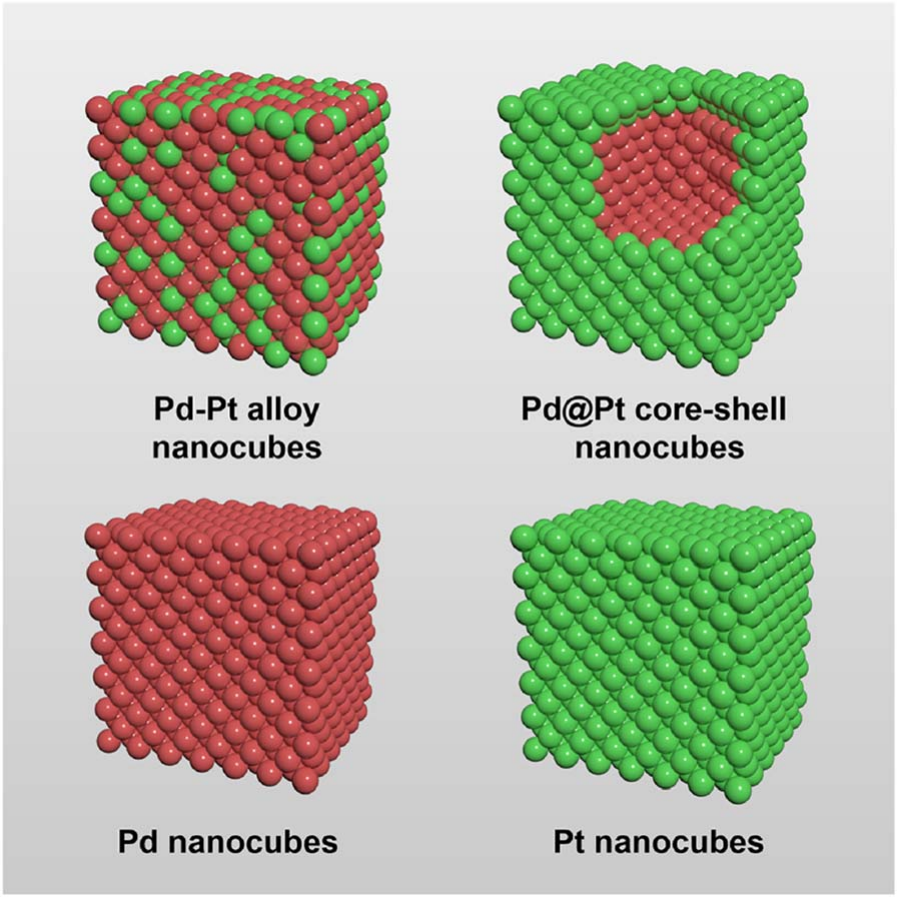
Geometrical illustration of the Pd–Pt alloy, Pd@Pt core–shell, Pd, and Pt nanocubes. Red and green denote Pd and Pt, respectively.

Herein, we prepared Pd–Pt alloy, Pd@Pt core–shell, Pd, and Pt nanocubes with identical {100} facets and similar edge size to investigate the effect of atomic distribution in Pd–Pt bimetallic NCs for electrochemical MOR (Fig. 1). The four types of nanocubes have different atomic distributions. The surface of Pd–Pt alloy nanocubes is composed of both Pd and Pt atoms, while Pd@Pt core–shell nanocubes have a Pt surface and a Pd core region. Unlike the bimetallic Pd–Pt alloy and Pd@Pt core–shell nanocubes, Pd and Pt nanocubes are exclusively composed of pure Pd or Pt atoms, respectively. This distinct atomic distribution enables the study of compositional structure-dependent electrocatalytic performance. Indeed, the present work demonstrates that electrocatalytic activity of the Pd–Pt bimetallic nanocubes highly depends on their atomic distribution and combinations. Among the nanocubes, the Pd–Pt alloy nanocubes exhibited enhanced electrocatalytic activities than the Pd@Pt core–shell, Pt, and Pd nanocubes. Furthermore, Pd–Pt alloy nanocubes exhibited larger MOR activity than commercial Pt/C and Pd/C catalysts due to their optimal electronic structure. Apart from their superior MOR activity, the Pd–Pt alloy nanocubes also exhibited higher stability compared to the other nanocubes, as well as Pt/C and Pd/C catalysts. The controlled catalysis experiments unambiguously demonstrate that the activity and stability of the bimetallic NCs can be tuned by controlling their compositional structure.

## 2 Experiment

### 2.1 Chemical and materials

Chloroform (CHCl_3_, 99%, JUNSEI), chloroplatinic acid hexahydrate (H_2_PtCl_6_·6H_2_O, ≥99%, Sigma-Aldrich), glucose (C_6_H_12_O_6_, 99%, Sigma-Aldrich), hexadecyltrimethylammonium bromide (C_19_H_42_BrN, ≥99%, Sigma-Aldrich), l-ascorbic acid (C_6_H_8_O_6_, AA, Daejung), methanol (CH_3_OH, MeOH, 99.8%, Junsei), Nafion® perfluorinated resin solution (5 wt%, Sigma-Aldrich), *N*,*N*-dimethylformamide (DMF, 99.5%, Sigma-Aldrich), oleic acid (C_8_H_32_O_2_, OLA, 90%, Sigma-Aldrich), oleylamine (C_18_H_37_N, OAm, 70%, Sigma-Aldrich), commercial Pd/C (20 wt%, Alfa Aesar), commercial Pt/C (20 wt%, Alfa Aesar), poly(vinylpyrrolidone) (PVP, MW = 55 000, Sigma-Aldrich), potassium bromide (KBr, ≥99%, Sigma Aldrich), potassium chloride (KCl, ≥99%, Sigma Aldrich), potassium hydroxide (KOH, 95%, Samchun), potassium tetrachloroplatinate (K_2_PtCl_4_, >99.9%, Sigma-Aldrich), sodium tetrachloropalladate (Na_2_PdCl_4_, >99.9%, Sigma-Aldrich), sodium iodide (NaI, ≥99.99%, Sigma-Aldrich), and other chemicals were reagent grade and deionized water (DI) with a resistivity of greater than 18.3 MΩ cm was used in the preparation of reaction solutions.

### 2.2 Synthesis of Pd–Pt alloy nanocubes

In a typical synthesis of Pd–Pt alloy nanocubes, 1.5 mL of Na_2_PdCl_4_ (20 mM), 0.5 mL of K_2_PtCl_4_ (20 mM), 300 mg of NaI, and 160 mg of PVP were mixed with 10 mL of DMF in a 20 mL vial. After the vial had been capped, the mixture was ultrasonicated to get the homogeneous mixture. The mixture was transferred into a conventional oven and heated at 130 °C for 5 h before it was cooled to room temperature. The resulting colloidal products were collected by centrifugation and washed several times with an ethanol–DI water mixture.

### 2.3 Synthesis of Pd@Pt core–shell nanocubes

In a typical synthesis of Pd@Pt core–shell nanocubes, 1 mL of a suspension of the as-prepared Pd nanocubes was washed with DI water and re-dispersed in 1 mL of OAm. Then, 1 mL of the suspension of seed Pd nanocubes and 4 mL of OAm solution containing 100 mg of glucose (20 mg mL^−1^) and 5 mg of H_2_PtCl_6_·6H_2_O were added into a 30 mL vial, which connected with air through a syringe needle on the cap. The mixture was heated at 200 °C in an oil bath under magnetic stirring for 3 h, and then cooled down to room temperature. The final products were washed with mixture hexane–ethanol and finally dispersed in ethanol.

### 2.4 Synthesis of Pd nanocubes

In a typical synthesis of Pd nanocubes, 8.0 mL of an aqueous solution containing 105 mg of PVP, 60 mg of l-ascorbic acid, 270 mg of KBr, and 170 mg of KCl were placed in a 30 mL vial, and pre-heated in air under stirring at 100 °C for 10 min. Then, 3.0 mL of an aqueous solution containing 57 mg of Na_2_PdCl_4_ was added. After the vial had been capped, the reaction was maintained to process at 100 °C for 3 h. The product was collected by centrifugation and washed several times with DI water.

### 2.5 Synthesis of Pt nanocubes

In a typical synthesis of Pt nanocubes, 20 mg of Pt(acac)_2_ was dissolved in a mixed solvent containing 8 mL of OAm and 2 mL of OLA in water bath (60 °C) for 10 min, then dipped into oil bath which was preheated to 190 °C and stirred at 190 °C with a CO flow for 60 min. The resultant reaction mixture was then cooled down to room temperature. Pt nanocubes were precipitated out and washed twice with a mixture of toluene and ethanol. The precipitates were re-dispersed in ethanol.

### 2.6 Preparation of the working electrodes

All nanocubes were loaded onto a carbon support (Vulcan carbon XC-72) with a metal content of approximately 20%, relative to the total mass of Pd and/or Pt. To load the nanocubes onto the carbon support, a specific quantity of nanocubes and carbon support were dispersed in ethanol. Subsequently, the mixture was subjected to sonication for 5 h, followed by stirring overnight. Then, the carbon-supported metal nanocubes (catalyst/C) were collected *via* centrifugation, redispersed in 10 mL of acetic acid, and subjected to heating at 60 °C for 3 h to facilitate the removal of chemical species adsorbed onto the surface of the metal nanocubes. Finally, the catalyst/C was retrieved through centrifugation, rinsed six times with ethanol, and then dried overnight in an oven. For the preparation of catalyst inks, catalyst/C (2.5 mg), isopropyl alcohol (0.50 mL), and Nafion solution (10 μL, 5 wt%) were added in deionized water (2.00 mL), followed by sonication for 30 min. In this study, a binder/ionomer, Nafion® perfluorinated resin solution from Sigma-Aldrich, was employed. The commercial Pd/C and Pt/C (20 wt%) catalysts were sourced from Alfa Aesar. The procedure for preparing the commercial catalyst was also analogous to that of the metal nanocube catalyst, involving the formulation of a catalyst ink. For the preparation of the electrode, the glassy carbon electrode (GCE) was polished using 0.05 μm Al_2_O_3_ and subsequently washed with deionized (DI) water before utilization. The GCE (diameter: 5 mm) featured a geometric area of 0.196 cm^2^. Subsequently, 10.0 μL of the resultant catalyst ink was carefully deposited onto a GCE and allowed to dry within an oven. To ensure uniformity, we took measures to maintain a consistent total catalyst mass of 5.1 μg cm^−2^ on the GC electrode.

### 2.7 Electrochemical measurement

Electrochemical measurements were carried out using a Bio-logic EC-Lab SP-300 in a three-electrode setup. The counter electrode was Pt wire, and the reference electrode was Hg/HgO (1 M NaOH). The GCE was used as a working electrode. The 0.1 M KOH and 0.5 M methanol were used as electrolyte and fuel for MOR experiment. All experiments were conducted at room temperature. The GCE was electrochemically cleaned through 50 potential cycles between −0.900 and 0.250 V *vs.* Hg/HgO at a scan rate of 50 mV s^−1^ in 0.1 M KOH. The electrolyte solutions were purged with Ar-gas for 30 min before the experiments. For the MOR, CVs for all catalysts were recorded between −0.900 and 0.250 V *vs.* Hg/HgO at a scan rate of 50 mV s^−1^ in both 0.1 M KOH and 0.1 M KOH + 0.5 M methanol. For stability testing using CAs, the potential was set at the oxidation peak potential of each catalyst and monitored for 5000 s. CO-stripping experiments involved saturating the catalyst-loaded GCE surface with CO by purging CO gas in 0.1 M KOH while maintaining the working electrode at −0.3 V *vs.* Hg/HgO for 15 min. Then, CO dissolved in the electrolyte was removed by purging with Ar-gas for 40 min. CO-stripping experiments were carried out between −0.900 and 0.250 V *vs.* Hg/HgO at a scan rate of 50 mV s^−1^. Electrochemically active surface area (ECSA) was determined using the equation: ECSA = *Q*_o_/*q*_o_, where *Q*_o_ represents the surface charge from the area below the oxygen reduction CV curve, and *q*_o_ is associated with the charge required for the oxygen monolayer reduction on Pd (420 μC cm^−2^) and Pt (424 μC cm^−2^).

### 2.8 Characterization

Transmission electron microscopy (TEM) and scanning electron microscopy (SEM) images of the prepared Pd–Pt alloy, Pd@Pt core–shell, Pd, and Pt nanocubes were obtained on Jeol JEM-2100F and Jeol JEM-7210F, respectively. Inductively coupled plasma-optical emission spectrometry (ICP-OES) measurement was carried out using a Spectroblue-ICP-OES (Ametek). X-ray diffraction (XRD) measurements were conducted on a Rigaku D/MAX2500V/PC scanning for 2*θ* at 30 to 90°. X-ray photoelectron spectroscopy (XPS) measurements were conducted on a Thermo-Fisher K-alpha. SEM analysis were done using instruments at total-period analysis center for Ulsan chemical industry of the Korea Basic Science Institute (KBSI).

## 3 Results and discussion

### 3.1 Synthesis and characterization of the nanocubes

**Fig. 2:**
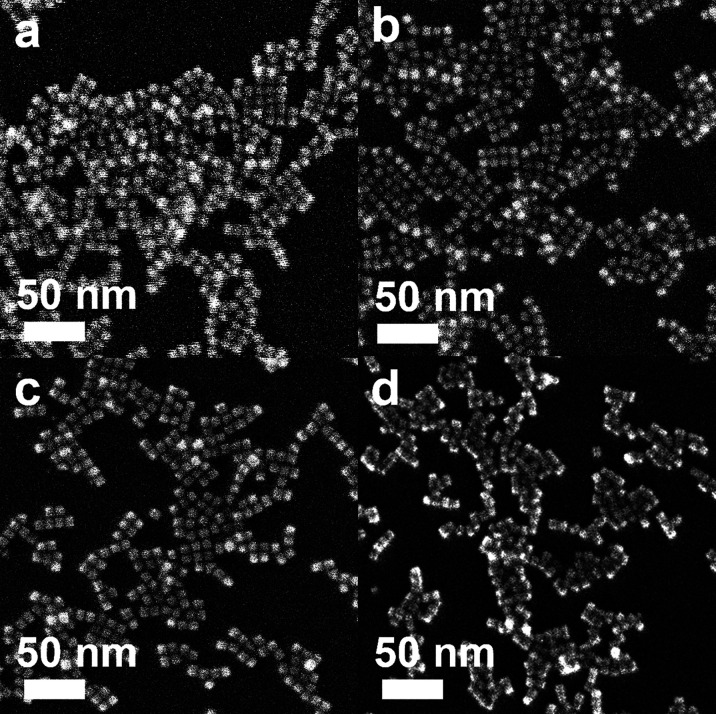
SEM images of (a) Pd–Pt alloy, (b) Pd@Pt core–shell, (c) Pd, and (d) Pt nanocubes.

**Fig. 3:**
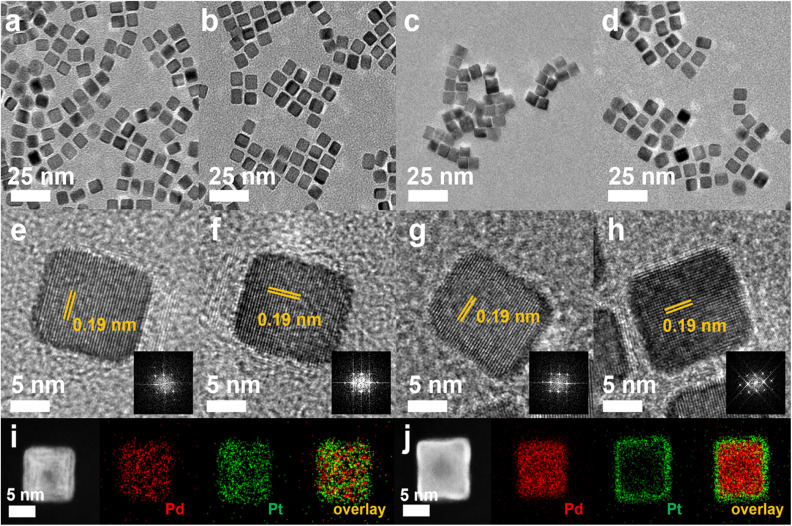
Structure characterization of Pd–Pt alloy, Pd@Pt core–shell, Pt and Pd nanocubes. (a–d) TEM, and (e–h) HR-TEM images including the correspond FFT images. HAADF-STEM images and corresponding EDS elemental mapping analysis of (i) Pd–Pt alloy and (j) Pd@Pt core–shell nanocubes.

The nanocubes with different compositional structures of Pd and Pt were synthesized using a wet-chemical synthesis method, following the previously reported procedure with a slight modification.^38–41^ The Pd–Pt alloy nanocubes were synthesized by co-reduction of Pd and Pt precursors in a reaction mixture containing NaI, PVP, and DMF, respectively, following the previous reported.^40^ On the other hand, the synthesis of bimetallic Pd@Pt core–shell nanocubes was accomplished through epitaxial growth of the Pt shell on the pre-synthesized Pd nanocubes with an edge length greater than 8 nm.^41^ The utilization of different chemicals and reaction temperature enables the formation of {100}-faceted nanocubes with different atomic distribution by providing optimal Pd and Pt growth rate. SEM images in Fig. 2 depict the prepared nanocubes, highlighting their uniform cubic morphology and similar sizes across different samples. TEM images of the products further confirm the well-defined cubic shape of the products, with average edge lengths of 8.4 ± 1.6 nm, 9.2 ± 1.6 nm, 8.3 ± 1.4 nm, and 8.7 ± 1.1 nm for Pd–Pt alloy, Pd@Pt, Pd, and Pt nanocubes, respectively (Fig. 3a–h). The adjacent lattice fringes of all nanocubes exhibit *d*-spacings around 0.194–0.196 nm, corresponding to the (200) planes of face-centered cubic (fcc) Pd–Pt alloy, Pd, and Pt. This confirms the successful formation of identical (100) faceted nanocubes (Fig. 3e–h). Furthermore, the highly crystalline nature of the prepared nanocubes was confirmed by the fast Fourier transform (FFT) patterns obtained from a single Pd–Pt alloy, Pd@Pt, Pd, and Pt nanocubes (inset in Fig. 3e–h).

**Fig. 4:**
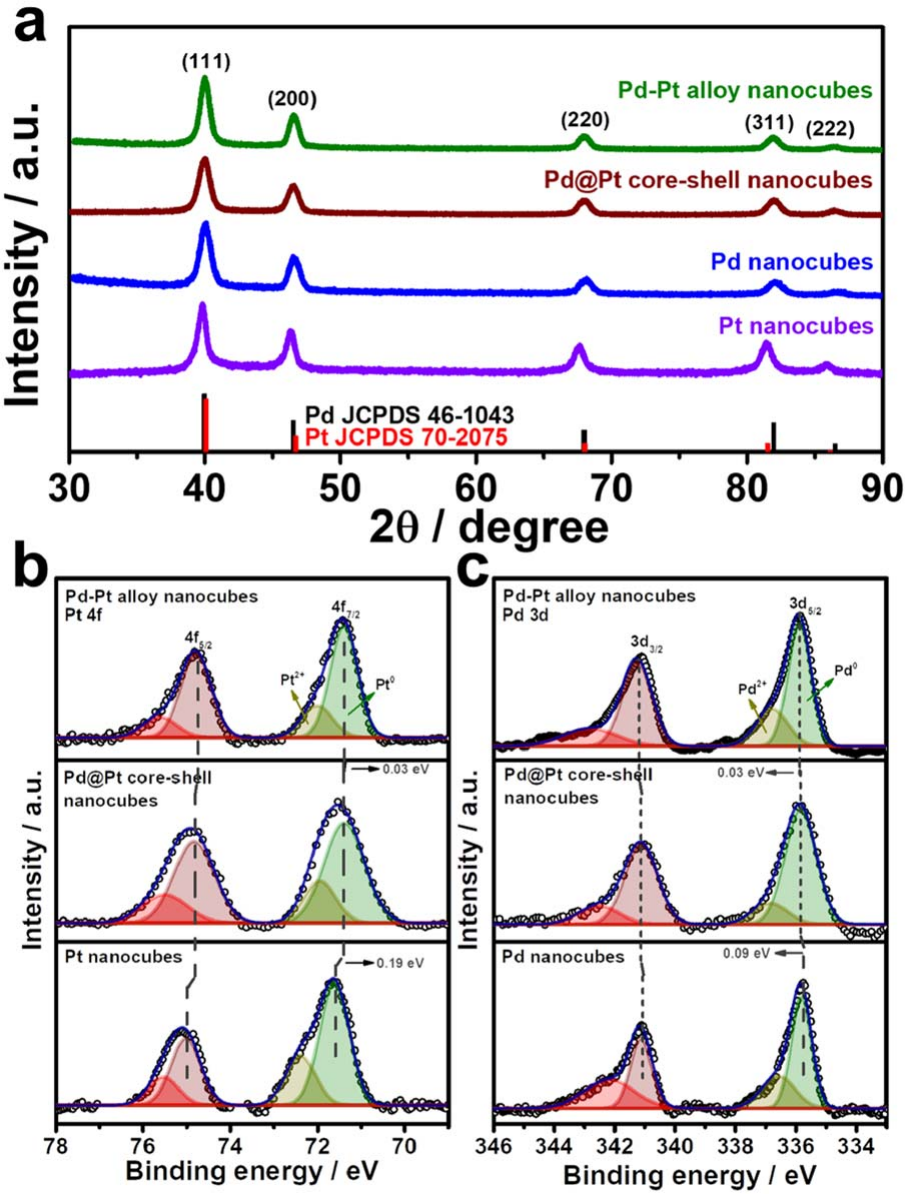
(a) XRD patterns of Pd–Pt alloy, Pd@Pt core–shell, monometallic Pd and Pt nanocubes. The red and black bars are referred to Pt and Pd JCPDS card, respectively. XPS spectra of (b) Pd 3d and (c) Pt 4f of bimetallic Pd–Pt alloy, Pd@Pt core–shell, Pd, and Pt nanocubes, respectively.

To investigate the compositional structure of the Pd–Pt bimetallic nanocubes, including Pd–Pt alloy and Pd@Pt nanocubes, high-angle annular dark-field scanning transmission electron microscopy-energy dispersive X-ray spectroscopy (HAADF-STEM-EDS) measurements were conducted. Fig. 3i and S1† exhibit the uniform distribution of Pd and Pt signals throughout the entire nanocube, indicating the successful formation of the Pd–Pt alloy compositional structure. The Pd/Pt atomic ratio in the Pd–Pt alloy nanocubes, measured using EDS and ICP-OES measurements, was determined to be 3 : 1. In contrast, the HAADF-STEM-EDS images shown in Fig. 3j show that Pd signals are predominantly observed in the core region, while Pt signals are clearly detected in the surface shell region, demonstrating the successful formation of Pd@Pt core–shell nanocubes. The Pd/Pt atomic ratio in the Pd@Pt core–shell nanocubes, as determined by EDS and ICP-OES measurements, was also found to be 3 : 1, matching the ratio observed in the Pd–Pt alloy nanocubes (Fig. S1 and Table S1†). The crystal structures of the nanocubes were investigated using XRD patterns. Fig. 4a and S2† displays the XRD patterns of the nanocubes, revealing all the nanocubes possess characteristic diffraction peaks corresponding to the fcc crystal structure. Comparing the diffraction peak positions, pure Pt nanocubes exhibit slightly negative-shifted peak positions compared to those of Pd nanocubes, which can be attributed to the slightly larger atomic size of Pt atoms compared to Pd atoms (Fig. S2†).^42–44^ The lattice distance from XRD pattern also well-match with the TEM images that four different types of Pd–Pt nanocubes (Table S2†). In the case of the Pd–Pt alloy and Pd@Pt core–shell nanocubes, the diffraction peak positions locate between those of pure Pt and Pd nanocubes. This observation further supports their Pd–Pt bimetallic nature, indicating the successful formation of the Pd–Pt alloy and Pd@Pt core–shell compositional structures. To investigate the surface composition and chemical states of the nanocubes, XPS analysis was conducted (Fig. 4b, c, S3, Tables S3 and S4†). In the XPS spectra of the Pt 4f core levels, the binding energies of 71.36 and 74.72 eV were observed for the Pd–Pt alloy nanocubes, corresponding to Pt 4f_7/2_ and 4f_5/2_, respectively. These positions are negatively shifted compared to those of the Pd@Pt core–shell (71.39 and 74.79 eV) and Pt nanocubes (71.58 and 75.00 eV) (Fig. 4b). The Pt 4f binding energy order of the nanocubes follows the sequence: Pt nanocubes > Pd@Pt core–shell nanocubes > Pd–Pt alloy nanocubes. The binding energies of Pd 3d_5/2_ and 3d_3/2_ for the Pd–Pt alloy nanocubes (335.88 and 341.17 eV) shifted to higher values compared to those of the Pd@Pt core–shell (335.85 and 341.12 eV) and Pd nanocubes (335.76 and 341.08 eV) (Fig. 4c). These XPS results indicate the electron transfer from Pd to Pt in both bimetallic Pd–Pt alloy and Pd@Pt core–shell nanocubes. Furthermore, the subtle differences in binding energies between the Pd–Pt alloy nanocubes and Pd@Pt core–shell nanocubes indicate distinct interplay between Pd and Pt atoms, which is contingent upon the compositional structure. These findings emphasize that despite having an identical Pd/Pt atomic ratio, the Pd–Pt alloy and Pd@Pt core–shell nanocubes exhibit different electronic structures, leading to unique electrocatalytic properties.

### 3.2 Electrocatalytic MOR performance

**Fig. 5:**
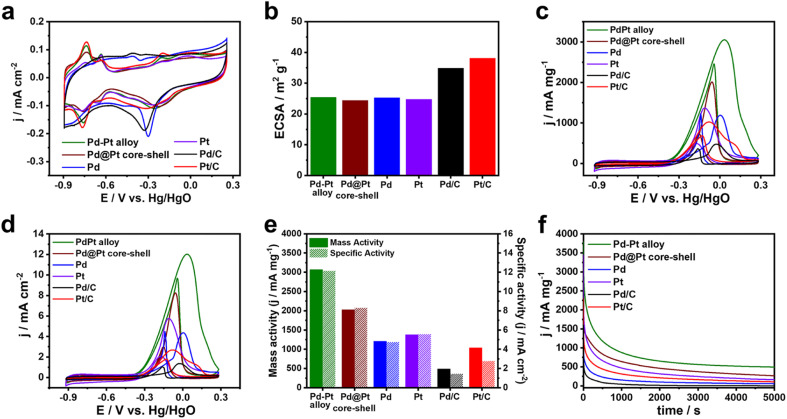
Electrocatalytic MOR properties of Pd–Pt alloy, Pd@Pt core–shell, Pt and Pd nanocubes, including commercial Pt/C and Pd/C in 0.1 M KOH and 0.5 M MeOH at 50 mV s^−1^. (a) CVs curves in 0.1 M KOH and (b) ECSAs of different catalysts. (c) Mass-normalized and (d) ECSA-normalized CV curves of different catalysts. (e) Mass and specific activity of different catalysts. (f) CA curves of different catalysts for 5000 s.

To examine the influence of compositional structure on the electrocatalytic reaction, the catalytic performances of the prepared Pd–Pt alloy, Pd@Pt core–shell, Pd, and Pt nanocubes were evaluated for MOR and compared with those of commercial Pd/C and Pt/C catalysts. Cyclic voltammograms (CVs) of the Pd–Pt alloy, Pd@Pt core–shell, Pd, and Pt nanocubes were obtained in a three-electrode system using a scan rate of 50 mV s^−1^ in 0.1 M KOH electrolyte (Fig. 5a). The catalysts displayed the hydrogen adsorption/desorption peak within the range of −0.9 to −0.6 V *vs.* Hg/HgO, along with the metal oxide reduction peak spanning −0.45 to 0.0 V *vs.* Hg/HgO (Fig. 5a).^45–47^ Compared with Pd nanocubes and Pd/C, the catalysts containing Pt surface atoms such as Pd–Pt alloy, Pd–Pt alloy nanocubes, Pd@Pt nanocubes, Pt nanocubes, and Pt/C exhibited metal oxide reduction peak at more positive potential.^48,49^

The ECSAs of the catalysts were calculated from the oxygen species desorption regions in the CVs. The ECSAs of the Pd–Pt alloy, Pd@Pt core–shell, Pd, and Pt nanocubes were determined to be 25.9, 24.4, 25.3, and 24.7 m^2^ g^−1^, respectively (Fig. 5b). The similar ECSAs of the nanocubes can be attributed to their comparable sizes, enabling investigation of the compositional effect on electrocatalytic activity independent of surface area. The ECSAs of the Pd/C and Pt/C catalysts were 34.9 and 38.1 m^2^ g^−1^, respectively, reflecting their smaller sizes (Fig. 5b). Fig. 5c and d shows the CVs of MOR obtained using the different catalysts in 0.1 M KOH solution containing 0.5 M methanol. The current measured in the CVs was normalized to the mass of the catalysts loaded on the GCE and the ECSA, representing the mass activity and specific activity, respectively. The Pd–Pt alloy nanocubes exhibit the highest mass activity, with a value of 3070 mA mg^−1^, which is 1.5, 2.5, 2.2, 6.3, and 3.0 times higher than the those of the Pd@Pt core–shell nanocubes (2030 mA mg^−1^), Pd nanocubes (1210 mA mg^−1^), Pt nanocubes (1380 mA mg^−1^), Pd/C (490 mA mg^−1^), and Pt/C (1040 mA mg^−1^) catalysts, respectively (Fig. 5c and e). Despite the lower mass activity compared to the Pd–Pt alloy nanocubes, the Pd@Pt core–shell nanocubes exhibit 1.7 and 1.5 times higher mass activity compared to pure Pd and Pt nanocubes, respectively. In addition, the similar trend across the catalysts was also observed in the specific activity (Fig. 5d and e). These results demonstrate the positive effect by combination of Pd–Pt bimetallic composition. Moreover, the higher MOR activities observed in all the nanocubes compared to commercial Pt/C and Pd/C indicate that the {100} surfaces of both Pt and Pd are more effective for electrochemical methanol oxidation than the mixed surface of commercial Pt/C and Pd/C catalysts.^15,50–53^

**Fig. 6:**
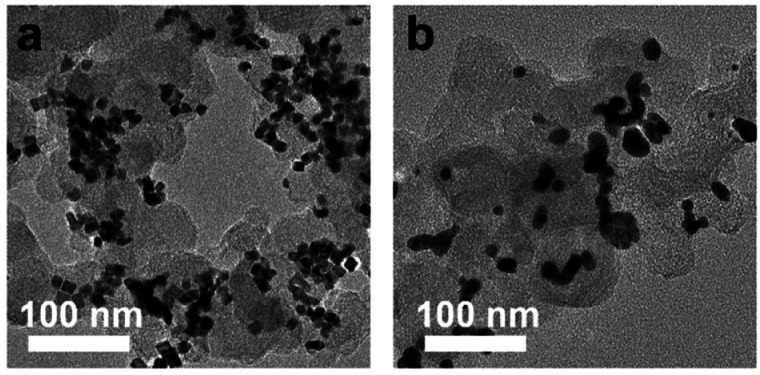
The TEM images of (a) Pd–Pt alloy nanocubes and (b) Pt/C after CAs test.

To assess the stability of these catalysts, chronoamperometry (CA) curves of all nanocubes were obtained and compared with those of Pd/C and Pt/C catalysts. As shown in Fig. 5f, the current densities of monometallic catalysts such as Pd nanocubes, Pt nanocubes, Pd/C, and Pt/C rapidly dropped and their current densities were 108, 168, 16, and 51 mA mg^−1^ after 5000 s, respectively, suggesting the poor MOR stabilities. In contrast, the current density of Pd–Pt alloy and Pd@Pt core–shell nanocubes could maintain above 521 and 256 mA mg^−1^ after 5000 s, respectively. Notably, Pd–Pt alloy nanocubes exhibited superior stability for MOR compared to Pd@Pt core–shell nanocubes. To check the excellent stability of the Pd–Pt alloy nanocubes, morphology and composition changes were investigated using TEM measurements. A TEM image shown in Fig. 6a demonstrates that the morphology of the Pd–Pt alloy nanocubes is preserved even after the stability test. Furthermore, the Pd/Pt atomic ratio, as determined through EDS analysis, maintained a high degree of similarity to its initial value of 3 : 1 throughout the experiment, with a measured ratio of 75.81 : 24.19. In contrast, aggregated morphology of Pt/C after stability test was observed in TEM image (Fig. 6b). These results provide evidence that the sustained activity of the Pd–Pt alloy nanocubes can be attributed to the preservation of their structural and compositional integrity.

**Fig. 7:**
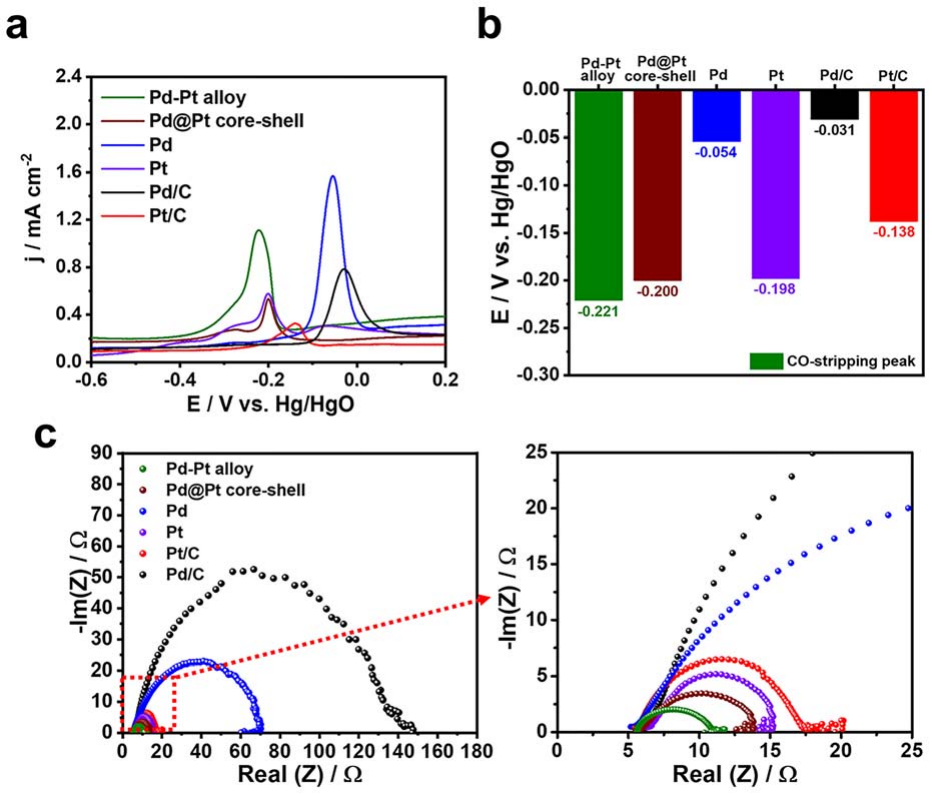
(a) CO-stripping measurements of different catalysts and (b) corresponding maximum current potentials. (c) Nyquist plots of different catalysts in 0.1 M KOH at 50 mV s^−1^.

To gain insights into the substantial enhancement in the MOR performance of the Pd–Pt alloy nanocubes compared to the Pd@Pt core–shell nanocubes and other monometallic catalysts, a CO stripping experiment was conducted. This experiment was performed because the strong bonding between the catalyst surface and CO, which is generated during electrochemical methanol oxidation, can hinder MOR by blocking the adsorption of methanol on catalyst active sites. Hence, an efficient MOR catalyst should have the ability to efficiently remove or scavenge the CO adsorbed on the catalyst surface. In Fig. 7a and b, the bimetallic Pd–Pt alloy and Pd@Pt core–shell nanocubes exhibited an maximum current potential of −0.221 and −0.200 V, respectively, for the oxidative elimination of CO to CO_2_. These maximum current potentials were observed at more negative potentials compared to those of the Pt nanocubes (−0.198 V), Pd nanocubes (−0.054 V), Pt/C (−0.138 V), and Pd/C (−0.031 V), respectively (Fig. 7a and b). Importantly, the higher CO removal capability of the Pd–Pt alloy nanocubes compared to that of the Pd@Pt core–shell nanocubes, as well as other catalysts, can account for their enhanced MOR activity and stability. Furthermore, the electrochemical resistant of the catalysts measured by electrochemical impedance spectroscopy (EIS) analysis. In Fig. 7c, it can be observed that bimetallic Pd–Pt nanocubes, including Pd–Pd alloy and Pd@Pt core–shell nanocubes, exhibited smaller arc radius compared to monometallic Pd or Pt catalysts. This finding indicates that the formation of a Pd–Pt bimetallic compositional structure is advantageous in enhancing their electron transfer ability compared to monometallic Pd or Pt compositions. Notably, smaller arc radius of Pd–Pd alloy nanocubes compared to Pd@Pt core–shell nanocubes implies the Pd–Pt alloy is more efficient than Pd@Pt core–shell structure to transfer electron. Consequently, the enhanced MOR activity and stability of the Pd–Pt alloy nanocubes, compared to the Pd@Pt core–shell nanocubes and monometallic Pd or Pt catalysts, can be attributed to their superior abilities in CO removal and electron transfer.

## 4 Conclusions

In conclusion, we have successfully demonstrated that the electrocatalytic activity of Pd–Pt based nanocubes for the MOR is intricately tied to the atomic distribution of Pd and Pt. This has been accomplished through a meticulous investigation involving Pd–Pt alloy, Pd@Pt core–shell, Pt, and Pd nanocubes. Notably, in comparison to Pd@Pt core–shell, Pt, and Pd nanocubes with monometallic surfaces, the Pd–Pt alloy nanocubes showed significantly enhanced catalytic activity, achieving an impressive mass (specific) activity of approximately 3070 mA mg^−1^ (12.17 mA cm^−2^). Furthermore, the stability of the Pd–Pt alloy nanocubes was superior to that of Pd@Pt core–shell, Pt, and Pd nanocubes. The exceptional electrocatalytic activity and stability of the Pd–Pt alloy nanocubes can be ascribed to their adeptness at CO removal and the modification of surface electronic structure, a consequence of the interplay between Pd and Pt within the alloy phase. These findings hold promise for applications in other electrochemical systems, with potential for even further enhancement of catalytic performance through the strategic manipulation of atomic distribution in nanostructures.

## Author contributions

Respati K. Pramadewandaru: investigation, writing – original draft. Young Wook Lee: analysis, writing – editing. Jong Wook Hong: supervision, funding, acquisition, conceptualization, writing – editing.

## Conflicts of interest

There are no conflicts to declare.

## Supplementary Material

RA-013-D3RA04837C-s001
