# Supplementary material for: Synergistic effect of bimetallic Pd–Pt nanocrystals for highly efficient methanol oxidation electrocatalysts
Source: RSC Adv. 2023 Sep 8;13(39):27046–53. doi: 10.1039/d3ra04837c (PMC10486200; doi:10.1039/d3ra04837c)
Supplement: RA-013-D3RA04837C-s001 [file RA-013-D3RA04837C-s001.pdf]

Electronic Supplementary Material (ESI) for RSC Advances.
This journal is © The Royal Society of Chemistry 2023

Supporting Information

Synergistic Effect of Bimetallic Pd-Pt Nanocrystals for the Highly Efficient Methanol Oxidation Electrocatalysts

Respati K. Pramadewandaru,[a] Young Wook Lee,*[b] and Jong Wook Hong*[a]

[a]*Department of Chemistry, University of Ulsan, Ulsan 44776, Korea. Email: jwhong@ulsan.ac.kr*
[b]*Department of Education Chemistry and Research Institute of Natural Sciences, Gyeongsang National University, Jinju 52828, Republic of Korea. Email: lyw2020@gnu.ac.kr*

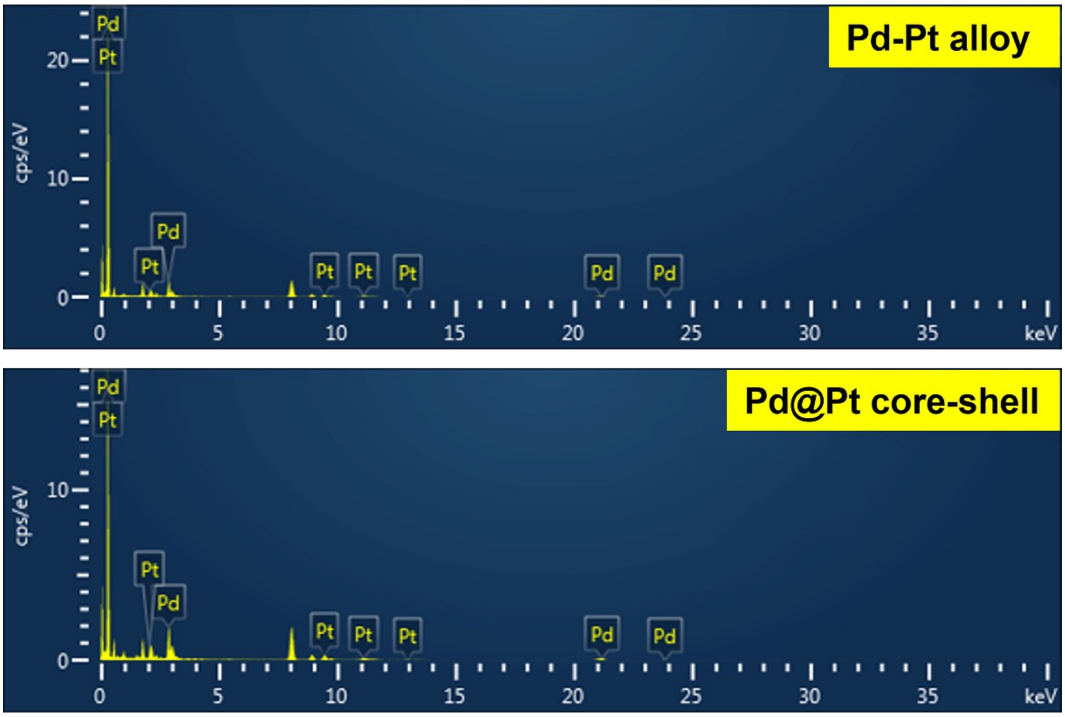

Fig. S1. EDS spectra of (a) Pd-Pt alloy and (b) Pd@Pt core-shell nanocubes.

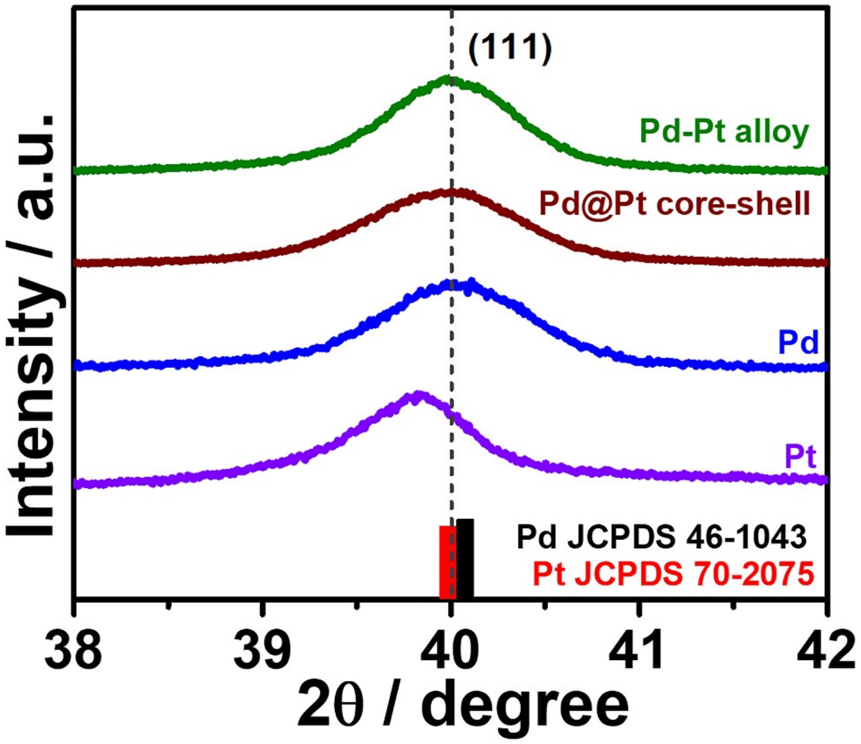

Fig. S2. The high-magnification XRD pattern of Pd-Pt alloy, Pd@Pt core-shell, Pd, and Pt nanocubes.

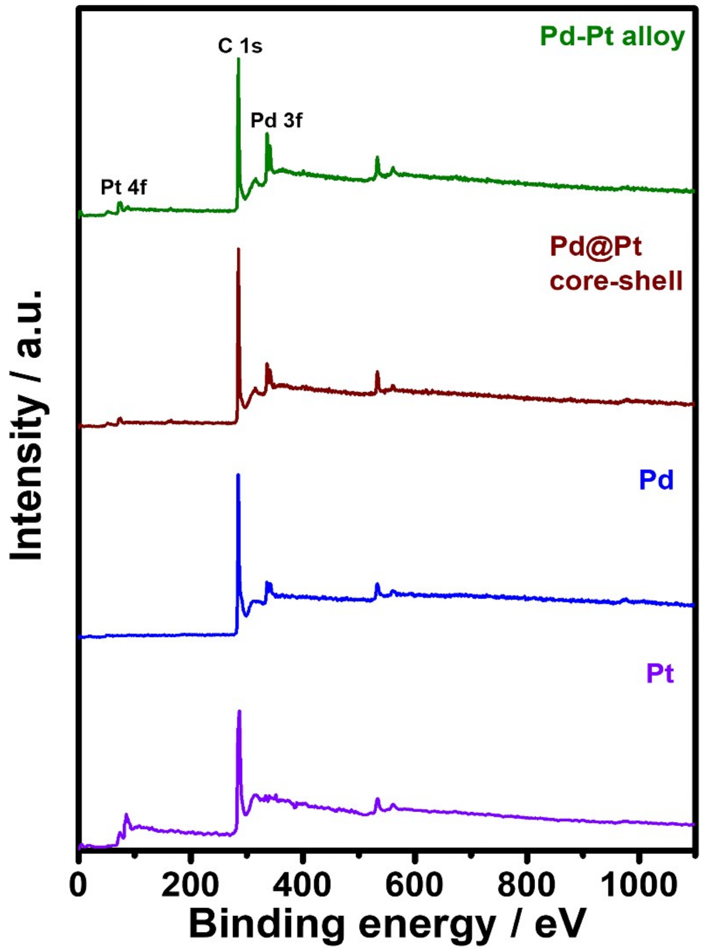

Fig. S3. The XPS survey spectra of Pd-Pt alloy, Pd@Pt core-shell, Pd, and Pt nanocubes.

Table S1. The comparison of EDS and ICP-OES results for Pd-Pt alloy and Pd@Pt core-shell nanocubes.

**Catalyst**	**EDS Element**		**ICP-OES**	
	**Pd**	**Pt**	**Pd**	**Pt**
**Pd-Pt alloy**	**76.20**	**23.80**	**74.33**	**25.67**
**Pd@Pt core-shell**	**72.03**	**27.97**	**72.88**	**27.12**

Table S2. Table as compared the lattice distance from XRD pattern and TEM images of different nanocubes.

Catalyst	XRD pattern	TEM image
**PdPt alloy**	1.95 Å	1.95 Å
**Pd@Pt core-shell**	1.95 Å	1.95 Å
**Pd**	1.94 Å	1.94 Å
**Pt**	1.96 Å	1.96 Å

Table S3. XPS spectra of Pt 4f core levels for nanocubes with different atomic distribution.

Catalyst	Pt $4f_{5/2}$		Pt $4f_{7/2}$	
	Pt^{2+}	Pt^{0}	Pt^{2+}	Pt^{0}
**Pd-Pt alloy**	75.51 eV	74.72 eV	71.99 eV	71.36 eV
**Pd@Pt core-shell**	75.52 eV	74.79 eV	71.92 eV	71.39 eV
**Pt**	75.55 eV	75.00 eV	72.37 eV	71.58 eV
**Pd**	-	-	-	-

Table S4. XPS spectra of Pd 3d core levels for nanocubes with different atomic distribution.

Catalyst	Pd $3d_{3/2}$		Pd $3d_{5/2}$	
	Pd^{2+}	Pd^{0}	Pd^{2+}	Pd^{0}
**Pd-Pt alloy**	342.90 eV	341.17 eV	336.76 eV	335.88 eV
**Pd@Pt core-shell**	342.57 eV	341.12 eV	336.90 eV	335.85 eV
**Pd**	342.18 eV	341.08 eV	336.48 eV	335.76 eV
**Pt**	-	-	-	-
